# The silent trauma: U.S. immigration policies and mental health

**DOI:** 10.1016/j.lana.2025.101048

**Published:** 2025-03-01

**Authors:** Omid Dadras, Mohammad Sediq Hazratzai

**Affiliations:** a Research Centre for Child Psychiatry, University of Turku, Turku, Finland; b Center for Immigrant and Refugee Health (CIRH), Public Health Institute (PHI), Sacramento, CA, USA

In January 2025, President Trump issued an executive order suspending the U.S. Refugee Admissions Program (USRAP), citing national security concerns.^1^ This policy shift, alongside stricter asylum policies and expanded immigration enforcement, has exacerbated fear, insecurity, and anxiety among migrant and refugee populations. The indefinite suspension of USRAP affects thousands of asylum seekers, including Afghan allies and families awaiting reunification, leaving many in precarious conditions with heightened mental health risks.^2^

Furthermore, the reinstatement of the ‘Remain in Mexico’ policy now mandates asylum seekers to await U.S. court hearings outside the country, effectively reinstating family detention protocols.^1^ Previous studies have demonstrated that immigration detention is associated with higher rates of depression, post-traumatic stress disorder (PTSD), and suicidal ideation, particularly among children.^3^ Adolescents who have had a family member detained or deported within the past year demonstrate higher odds of suicidal ideation, alcohol use, and clinical externalising behaviours.^4^ Children of detained or deported parents show higher rates of PTSD symptoms, internalising problems, and overall functioning issues compared to those whose parents have stable immigration status.^5^ The effects persist after release, with longer periods post-detention associated with more depressive symptoms.^3^

The expansion of expedited removal procedures allows for the rapid deportation of individuals unable to prove continuous U.S. residence for the past two years.^6^ Policies like these intensify psychological distress in undocumented communities, leading to chronic fear of deportation, family separations, and barriers to accessing healthcare and legal protections.^7^ Migrants, especially unaccompanied minors, experience compounded mental health challenges due to pre-migration trauma, prolonged uncertainty, and structural exclusion, increasing risks of PTSD, depression, and social withdrawal.^8^

In addition, the administration's efforts to redefine birthright citizenship, aiming to exclude children of undocumented immigrants from automatic U.S. citizenship, introduce significant maternal mental health risks. The fear of statelessness for their child, coupled with deportation concerns, heightens prenatal stress, anxiety, and depression—factors linked to preterm labour, low birth weight, and adverse neonatal outcomes.^9^ Beyond pregnancy, legal precarity disrupts early childhood attachment, placing these children at greater risk for PTSD, emotional dysregulation, and developmental delays. Studies on immigrant families show that chronic uncertainty and legal vulnerability contribute to poor long-term psychological and social outcomes.^10^

Restrictive immigration policies also have broader societal consequences, straining public health systems, social services, and community cohesion. The mental health burden of migration-related stress disproportionately affects racial and ethnic minority groups, exacerbating existing health inequities. The increased psychosocial distress among immigrant communities translates into greater demand for mental health services, yet systemic barriers to healthcare access persist. Many undocumented migrants avoid seeking care due to fear of deportation, resulting in delayed interventions and worsened health outcomes.^7^ Without intervention, these policies will widen disparities, increase healthcare costs, and hinder long-term integration efforts.

## Policy implications and the need for reform

Given the clear mental health risks associated with restrictive immigration policies, urgent policy reforms are needed. This includes:

1. Trauma-informed care in detention facilities, ensuring psychological support for detained individuals and families.
2. Mental health screenings for asylum seekers and newly arrived refugees to provide early intervention for PTSD and depression.
3. Community-based support services offering legal aid, counselling, and integration programmes to reduce psychological distress.
4. Protections for pregnant immigrants, ensuring access to prenatal care and mental health services to mitigate maternal and neonatal risks.
5. Ending family detention and expedited removal by replacing policies that separate families or detain children with community-based alternatives that prioritise family unity and psychological well-being.

The mental health impact of immigration policies extends far beyond individuals, affecting entire generations of migrant families and shaping the societal fabric of host communities.^9^ Research underscores that long-term exposure to immigration enforcement policies contributes to cumulative trauma, fostering intergenerational cycles of mental health disorders and legal precarity.^5^^,^^8^ While national security is a legitimate concern, evidence-based immigration policies must balance security with humanitarian and public health considerations. Without such reforms, current policies risk perpetuating trauma, worsening public health disparities, and undermining the principles of social justice, undermining the well-being of millions of migrants and their children. As the U.S. continues to redefine its immigration framework, policymakers must prioritise compassionate, evidence-based approaches that address the mental health needs of migrant populations while fostering long-term social cohesion and public health resilience.

## Contributors

**OD:** Supervision, conceptualisation, writing—original draft, review & editing.

**MSH:** Writing, review & editing.

## Declaration of interests

The authors declare no competing interests.
